# MicroRNAs Induce Epigenetic Reprogramming and Suppress Malignant Phenotypes of Human Colon Cancer Cells

**DOI:** 10.1371/journal.pone.0127119

**Published:** 2015-05-13

**Authors:** Hisataka Ogawa, Xin Wu, Koichi Kawamoto, Naohiro Nishida, Masamitsu Konno, Jun Koseki, Hidetoshi Matsui, Kozou Noguchi, Noriko Gotoh, Tsuyoshi Yamamoto, Kanjiro Miyata, Nobuhiro Nishiyama, Hiroaki Nagano, Hirofumi Yamamoto, Satoshi Obika, Kazunori Kataoka, Yuichiro Doki, Masaki Mori, Hideshi Ishii

**Affiliations:** 1 Department of Gastroenterological Surgery, Osaka University Graduate School of Medicine, Suita, Osaka, Japan; 2 Department of Frontier Science for Cancer and Chemotherapy, Osaka University Graduate School of Medicine, Suita, Osaka, Japan; 3 Department of Cancer Profiling Discovery, Osaka University Graduate School of Medicine, Suita, Osaka, Japan; 4 Faculty of Mathematics, Kyushu University, Fukuoka, Japan; 5 Division of Cancer Cell Biology, Cancer Research Institute of Kanazawa University, Kakuma-machi, Kanazawa, Japan; 6 Department of Bioorganic Chemistry, Osaka University Graduate School of Pharmaceutical Science, Suita, Osaka, Japan; 7 Department of Materials Engineering, The University of Tokyo, Bunkyo-ku, Tokyo, Japan; 8 Tokyo Institute of Technology, Chemical Resources Laboratory, Yokohama, Midori-ku, Japan; Centro Cardiologico Monzino, ITALY

## Abstract

Although cancer is a genetic disease, epigenetic alterations are involved in its initiation and progression. Previous studies have shown that reprogramming of colon cancer cells using Oct3/4, Sox2, Klf4, and cMyc reduces cancer malignancy. Therefore, cancer reprogramming may be a useful treatment for chemo- or radiotherapy-resistant cancer cells. It was also reported that the introduction of endogenous small-sized, non-coding ribonucleotides such as microRNA (miR) 302s and miR-369-3p or -5p resulted in the induction of cellular reprogramming. miRs are smaller than the genes of transcription factors, making them possibly suitable for use in clinical strategies. Therefore, we reprogrammed colon cancer cells using miR-302s and miR-369-3p or -5p. This resulted in inhibition of cell proliferation and invasion and the stimulation of the mesenchymal-to-epithelial transition phenotype in colon cancer cells. Importantly, the introduction of the ribonucleotides resulted in epigenetic reprogramming of DNA demethylation and histone modification events. Furthermore, *in vivo* administration of the ribonucleotides in mice elicited the induction of cancer cell apoptosis, which involves the mitochondrial Bcl2 protein family. The present study shows that the introduction of miR-302s and miR-369s could induce cellular reprogramming and modulate malignant phenotypes of human colorectal cancer, suggesting that the appropriate delivery of functional small-sized ribonucleotides may open a new avenue for therapy against human malignant tumors.

## Introduction

Every cancer cell is largely derived from stem or progenitor cells of normal somatic tissue via genetic and epigenetic alterations. These alterations inactivate growth-constraint tumor suppressor genes (TSGs) and activate growth-promoting oncogenes. Normal somatic cells are developed from a fertilized oocyte through an epigenetic program. Notably, the ectopic introduction of defined coding genes, OCT3/4, SOX2, KLF4, and c-MYC (OSKM), or OSK, which are exclusively expressed in embryonic stem cells (ESCs), induces full reprogramming of differentiated somatic cells back to pluripotent stem cells. We previously showed that the introduction of OSKM in epithelial cancer cells of gastrointestinal organs modulates the malignant phenotype. Our findings suggested that reprogramming can suppress cancer invasion, drug resistance, and tumorigenicity through the re-activation of the tumor suppressor p16INK4A pathway by demethylation of the promoter sequence [1]. Moreover, a recent mouse study of transgenic OSK factors showed that epigenetic modifications are involved in tumor initiation and development *in vivo*. This study also demonstrated that, in combination with the inactivation of TSG signals such as mutant Apc, the modifications can orchestrate epigenetic alterations by the polycomb group complex to the core module of histone H3 at lysine-27 [2]. Taken together with previous findings [1–6], epigenetic alterations, regardless of the causes or results of the genetic changes, contribute to the occurrence and development of malignancies and may be attractive for the discovery of novel therapeutic targets against human cancer.

Therapeutic applications that augment endogenous signaling pathways should have minimal toxicity *in vivo*. In this regard, drug discovery from endogenous microRNAs (miRNAs, miR), small-sized noncoding ribonucleotides (22 bp on average), are potential sources for innovative therapeutic strategies [7,8]. Because synthesized mature miRs do not integrate into the genome, they should exert their functions without undesired genomic integration events and, if combined with a drug delivery system, miRs could exert these specific effects on therapeutic targets [7,8].

Recent miR discovery studies have identified critical clusters, including miR-302s, which enhance the reprogramming effect in collaboration with viral-mediated transcription factor transfer [9–12]. It has been shown that a set of three miRs (miR-302s, miR-369s and miR-200c) selected from numerous miRs expressed exclusively in induced pluripotent stem cells (iPSCs)/ESCs, elicited reprogramming [13]; a significant role for miR-367 was also shown [12]. Reportedly, the introduction of miR-302s induced both cell cycle arrest by interfering with cyclin-dependent kinases (Cdks) and global demethylation of DNA in liver cancer and melanoma *in vitro* [4,5,14]. Here, we studied the effect of miR-302s and miR-369s *in vitro* and *in vivo*. These results suggested that cancer reprogramming using miR-302s and miR-369s may be an effective treatment option for cancer therapy.

## Materials and Methods

### Cell culture

Colorectal cancer cell lines HT29, DLD-1, SW480, HCT116, Caco2, Colo201, RKO, and Lovo cells and teratocarcinoma cell line NTERA (NTera 2/cl.D1 [NT2/D1]) were obtained from RIKEN (Tsukuba, Japan). These cells were cultured in RPMI-1640 medium (Nakalai Tesque, Kyoto, Japan) with 10% FBS (Gibco Life Technologies, Tokyo, Japan) and 500 μg/ml of penicillin-streptomycin.

### Transfection

Specific miRs and negative control (NC) miR used in *in vitro* and *in vivo* analysis were purchased (Gene Design Inc., Osaka, Japan; S1 Table). Cells were transfected with specific miRs and NC miR using lipofection (LP) or carbonate apatite (CA). In LP, cells were transfected with miRs using Lipofectamine iMax (Invitrogen, Darmstadt, Germany) according to the manufacturer’s protocol.

### Cell reprogramming

HT29 cells and DLD-1 cells were transfected with 10 nM of each miR using CA. Cells were incubated in RPMI-1640 with 10% FBS for 24 h and transfection was repeated every two days for a total of three times. After the third transfection, cells were seeded onto Matrigel-coated and mitomycin C-treated mouse embryonic fibroblasts (MEF). Cells were cultured in embryonal stem cell culture medium containing DMEM/F12 (Gibco Life Technologies, Tokyo, Japan), supplemented with 2 mM GlutaMAX, 20% knockout serum replacement (Gibco Life Technologies), 0.1 mM nonessential amino acids (NEAA, Gibco Life Technologies), 10 ng/ml basic fibroblast growth factor (bFGF, Wako, Tokyo, Japan), 55 μM 2-mercaptoethanol (Gibco Life Technologies), 1% penicillin-streptomycin, and chemical inhibitors, including 0.5 μM A83-01 (Stemgent, Cambridge, MA), 3 μM CHIR99021 (Stemgent), and 0.5 μM PD0325901 (Stemgent), at 37°C in a 5% CO_2_ incubator. Media was changed every two days and the cells were maintained at 37°C in a 21% CO_2_ incubator for an additional 21 days. During this period, these cancer cells were monitored for the formation of ES-like colonies. These were picked for further analysis with Alkaline Phosphatase (AP) Live Stain (500×) (Invitrogen) using an all-in-one fluorescence microscope (BZ-9000; Keyence, Osaka, Japan) with digital photographic capability for selection according to the manufacturer’s instructions. To study miRs transfection efficiency, DLD-1 cells were transfected with BLOCK-iT Alexa Fluorescent Control (Invitrogen) with CA or LP. In brief, seeded DLD-1 cells in a 6-well plate were transfected with BLOCK-iT Alexa Fluorescent Control and photographed after transfection using a Keyence BZ-8000 microscope. The fluorescence intensity of transfected cells as observed using a FACS BD FACSAria III cell sorter.

### Luciferase assay

The 3′ untranslated region (3′-UTR) of CDK2 was amplified by RT-PCR using the primers 5’-CTAGCTAGCTAGCCTTCTTGAAGCCCCCA-3' and 5’-CTAGCTAGCGAGCTACAAACTAAATTACA-3'. Primers were subcloned, ligated into the pmirGLO Dual-Luciferase miRNA Target Expression Vector (Promega) using NheI, and confirmed by direct sequencing. Luciferase assays were conducted using 5 × 10^3^ DLD-1 cells plated in a 96-well plate. Cells were transfected using Lipofectamine 3000 (Invitrogen) in OptiMEM reduced serum media (Gibco) with 200 ng of empty vector or Luciferase-CDK2 3’UTR vector and either NC miR or miR-302s (final concentration, 25 nmol/L). Luciferase activity was measured 24 h post-transfection using the Dual-Glo Luciferase Assay System (Promega) according to the manufacturer’s protocol. Relative luciferase level was calculated as (Sample Luc/Sample Renilla)/(Control Luc/Control Renilla), where Luc is raw firefly luciferase activity and Renilla is the internal transfection control luciferase activity.

### Cell proliferation assay

To assess the proliferation and sensitivity of AP-positive ES-like colony-forming cancer cells to 5-fluorouracil (5-FU, Kyowa Hakko Kirin Co, Tokyo, Japan), 1×10^3^ cells were exposed to several concentrations of 5-FU for 72 h in a 96-well plate. Viable cells were evaluated using the Cell Counting Kit-8 (CCK-8) (Dojindo Molecular Technologies, Tokyo, Japan). To assess the influence of these indicated miRs on cell proliferation, HT29 cells and DLD-1 cells (5×10^4^ cells/well in a 12-well plate) were transfected with either miR-302s, miR-302s plus miR-369s, or NC miR, as described above. Cells were counted every day for three days, starting 24 h post-transfection.

### Cell cycle analysis

For cell cycle analysis by flow cytometry, 5×10^4^ DLD-1 cells were transfected with each miR in a 24-well plate, trypsinized after 72 h, washed with phosphate-buffered saline (PBS), and fixed in 70% ethanol on ice. After centrifugation, cells were stained with 50 mg/ml propidium iodide (PI) solution (Dojindo Molecular Technologies) and 0.1 mg/ml RNase A (Invitrogen) and analyzed by flow cytometry using a FACS BD FACSAria III cell sorter. Each histogram was constructed with data from at least 10,000 events and was used to calculate the percentage of the cell population in each phase.

### Cell invasion assay

Transwell invasion assays were carried out in 24-well modified chambers pre-coated with Matrigel (BD BioCoat, BD Biosciences, Franklin Lakes, NJ). DLD-1 cells and SW480 cells were transfected with either 10 nM of miR-302s, miR-369s, miR-302s plus miR-369s and NC miR using CA. After 48 h, cells were trypsinized and re-suspended at a concentration of 10×10^4^ cells/ml in serum-free medium, and 0.5 ml cell suspension was added to the top of each well. After 48 h incubation, cells migrating into the lower chamber, containing 10% FBS as a chemo-attractant, were fixed and stained with Wright-Giemsa stain (Diff-Quick; Sysmex, Kobe, Japan). Four random fields were counted in triplicate. Data are expressed as the median value with the standard error of the mean (s.e.m) of invaded cells in a relative ratio compared to NC miR-treated cancer cells.

### Cell differentiation study

To determine the differentiation ability of the generated ES-like colony-forming cancer cells *in vitro*, cells were cultured in floating cultivation to form embryoid bodies (EBs). After 4–5 days, EBs were transferred to 0.1% gelatin-coated plates and cultured in RPMI-1640 with 10% FBS for a further 14 days.

### RNA analysis

Total RNA was extracted using Trizol (Invitrogen, Tokyo, Japan). RNA quality was assessed with a NanoDrop ND-1000 spectrophotometer (NanoDrop Technologies, Wilmington, DE) at 260 and 280 nm (A260/280). Reverse transcription (RT) for miR was performed *in vitro* with the Taqman Reverse transcription microRNA Kit (Applied Biosystems, Tokyo, Japan). qPCR was performed with the universal Taqman PCR Master Mix (Applied Biosystems).

### RT-PCR

RNU48 expression in vitro and RNU6B expression in vivo were used as an internal control to determine the relative expression of miR. RT for miR was performed *in vitro* with miScript II RT Kit (Qiagen, Tokyo, Japan). qPCR was performed using the miScript SYBR Green PCR Kit (Qiagen). RNU6B expression was used as an internal control. Fold-changes were calculated and normalized using the CT method. mRNA levels were assayed using the Reverse Transcription System (Promega, Tokyo, Japan) and LightCycler FastStart Reaction Mix SYBR Green I in a Lightcycler (Roche, Tokyo, Japan). All results were normalized to a GAPDH control. RNA expression studies were independently repeated at least three times to confirm reproducibility.

### Protein analysis

Protein levels were determined by immunoblotting. Antibodies against Bak (1:200, Santa Cruz, Santa Cruz, CA), Bid (1:200, Santa Cruz), Bcl-2 (1:1000, CST, Tokyo, Japan), Bcl-xl (1:1000, CST), Mcl-1 (1:1000, CST), Caspase8 (1:1000, CST), Caspase3 (1:1000, CST), CyclinD1 (1:200, Santa Cruz), CDK2 (1:200, Santa Cruz), CDK4 (1:200, Santa Cruz), E-cadherin (1:1000, CST), vimentin (1:1000, CST), Zeb1 (1:1000, CST), Phospho-Rb (Ser807/811) (1:1000, CST), and the internal control ACTB (1:2000, Sigma Aldrich, Tokyo, Japan) were used.

### Immunoblotting

Cell pellets were lysed in ice cool radioimmunoprecipitation assay buffer with protease inhibitor. Protein concentrations were determined using the Bradford assay. Proteins were separated on 4–10% Mini-PROTEAN Precast Gels (BioRad, Tokyo, Japan). After overnight electrophoretic transfer onto PVDF membrane, membranes were blocked with Blocking One solution (Nacalai Tesque, Tokyo, Japan) for 1 h and incubated overnight at 4°C with the indicated primary antibody. Finally, membranes were incubated with secondary anti-mouse or-rabbit whole IgG coupled to horseradish peroxidase (GE Healthcare Life Sciences, Tokyo, Japan) for 1 h at room temperature. Antibody binding to target proteins was visualized by chemiluminescence using Amersham ECL Prime Western Blotting Detection Reagents (GE Healthcare Life Sciences).

### Immunocytochemistry

Cells were washed twice with PBS and fixed in 4% paraformaldehyde for 15 min at room temperature. Cells were then washed twice with PBS and permeated with 0.1% TritonX-100 for 15 min at room temperature. Unspecific binding was blocked by incubating in goat or horse serum (Vectastain, Funakoshi, Tokyo, Japan) for 10 min at room temperature. Cells were incubated with primary antibodies overnight at 4°C. Primary antibodies used were polyclonal rabbit anti-human Oct3/4 (1:400, MBL, Nagoya, Japan), polyclonal rabbit anti-human Sox2 (1:400, MBL), monoclonal mouse anti-human Nanog (1:2000, CST), monoclonal mouse anti-human Ki-67 Antigen (1:400, DAKO), and monoclonal rabbit anti-human E-cadherin (1:200, CST). The next day, cells were washed twice with PBS and treated with secondary antibody for 30 min at room temperature. Secondary antibodies used were anti-rabbit IgG (H+L), F(ab’)2 fragment (Alexa Fluorr 488 Conjugate, 1:1000, CST) or anti-mouse IgG (H+L), F(ab’)2 fragment (Alexa Fluorr 555 Conjugate) (1:1000, CST). Nuclei were stained with ProLong Gold Antifade reagent with DAPI (Invitrogen). Images were captured with a Keyence BZ-8000 microscope.

### DNA methylation analysis

miR302-transfected DLD-1 colorectal cancer cells were analyzed for DNA methylation. Briefly, cells were transfected with miR-302s or the mock control. Methylated proteins were immunoprecipitated from the cell lysate using methylation-binding protein. Samples were sequenced (Takara, Kyoto, Japan) to obtain whole genome-wide DNA methylation data. Data between the two samples were compared to determine the DNA methylation response to miR-302s transfection.

### Histone methylation analysis

Histone extraction and measurement of global methylation levels of histone 3 lysine-4 (H3K4) were performed using the Global Histone H3-K4 Methylation kit according to the manufacturer’s protocols (Abnova, Taipei, Taiwan).

### Mouse study

Animal studies were conducted in strict accordance with the principles and procedures approved by the Committee on the Ethics of Animal Experiments of Osaka University (approval number, 24-122-011). The tumorigenicity assay was performed using an *in vivo* xenograft model. First, 5 × 10^6^ cells suspended in a total volume of 200 μL DMEM/Matrigel (1:1 (v/v) suspension) were injected into the flanks of 7-8-week-old female mice (BALB/cAJcl-nu/nu). When tumor volumes reached 100 mm^3^, as measured with calipers and calculated using the formula V = (ab^2^)/2, where a is the length and b is the width, tumors were harvested and cut into 3–4 mm^3^ sections for serial transplantation. Sections were to confirm vasculaturization by immunohistochemistry using CD31, a vascular endothelial marker. Serially transplanted HT29 xenografts had the most abundant vasculature, was uniformly distributed over the whole tumor, and had the least amount of central necrosis. miRs/CA complexes were administered using a 30G needle via the tail vein. Tumor size and body weight were measured once every 3 days. Xenograft tumors and mouse blood were collected during sacrifice and preserved in 10% neutral-buffered formalin for histology and immunohistochemical studies or in RNAlater RNA Stabilization Reagent (Qiagen, Tokyo, Japan).

### Carbonate apatite preparation

To prepare a CA transfection mixture *in vitro*, 2 μg of each miR-302 (-a,-b,-c,-d), miR-369 (-3p, -5p) or NC miR was mixed with 4 μL of 1 M CaCl_2_ in 1 mL of serum-free bicarbonate (44 mM)-buffered DMEM medium (pH 7.5) incubated at 37°C for 30 min, and used for transfection [15–17]. This method was mainly used for *in vitro* experiments. For *in vivo* experiments, an inorganic solution (0.9 mM NaH_2_PO_4_, 1.8 mM CaCl_2_, pH 7.5) replaced the DMEM solution. For one mouse, a mixture of 15 μg of each miR was used for single injection. The solution was centrifuged at 12,000 rpm for 3 min and the pellet dissolved in 200 μl saline containing 0.5% albumin. Products in the solution were sonicated (38 kHz, 80 W) in a water bath for 10 min under 20°C to generate miR/CA complexes, which were injected intravenously within 5 min.

### Patient samples

Clinical samples were collected during surgery from nine patients undergoing surgical resection for colorectal cancer at Osaka University Hospital and its related hospitals in 2005. Patients are summarized in S2 Table. All patients were in agreement with the use of resected specimens in this study according to the guidelines approved by the Institutional Research Board (approved protocol #213).

### Immunohistochemical analysis

Immunohistochemical analysis was performed on 3.5 μm paraffin-embedded sections from xenografts. Paraffin-embedded sections were de-paraffinized in Hemo-De (Farma) and rehydrated in a graded ethanol series. Slides were heated in antigen retrieval buffer for 40 min, blocked with goat or horse serum for 20 min at room temperature, and incubated with monoclonal mouse anti-human Ki67 antigen (1:400, DAKO), polyclonal rabbit anti-human Oct3/4 (1:100, MBL), polyclonal rabbit anti-human Sox2 (1:200, MBL), or monoclonal mouse anti-human CK20 (DAKO) antibodies overnight at 4°C. The Vectastain ABC System (Vectastain, Funakoshi, Japan) was used to visualize antigen. Counter-staining was performed using hematoxylin.

### Immunofluorescence analysis

Immunofluorescence analysis was performed on 3.5 μm paraffin-embedded sections from xenografts to detect tumor vessels. Paraffin-embedded sections were de-paraffinized and treated as described for immunohistochemistry. Sections were blocked with goat serum for 20 min at room temperature and incubated with rat anti-mouse CD31 antibody (1:20, Dianova, Hamburg, Germany) overnight at 4°C. The next day, the secondary antibody anti-rat IgG (H+L), F(ab’)2 Fragment (Alexa Fluorr555 Conjugate) (1:1000, CST) was applied. Sections were counter-stained with ProLong Gold Antifade Reagent with DAPI. Images were captured with a Keyence BZ-8000 microscope.

### Alcian blue staining

Alcian blue staining was performed on 3.5 μm paraffin-embedded sections from xenografts to detect mucus. In brief, the slides were de-pareffinized in Hemo-De (Farma), rehydrated in a graded ethanol series, immersed in 3% acetic acid for 3 min, and stained in alcian blue solution (1 g Alcian blue 8GX, 3 ml acetic acid, and 97 ml distilled water) for 20 min. Sections were then washed and the nuclei counterstained with Kernechtrot (TCI, Tokyo, Japan) for 5 min.

### Apoptosis assay

The Annexin V-FITC Apoptosis Detection Kit (Biovision, Milpitas, CA) was used according to the manufacturer’s protocol for *in vitro* early and late phase detection of apoptotic cells. Briefly, to detect apoptosis *in vitro*, 20×10^4^ DLD-1 cells were transfected with miR-302s, miR-369s, miR-302s plus miR-369s or NC miR using CA in a 6-well plate in triplicate 60 h before the assay. Cells were trypsinized and analyzed by flow cytometry using a FACS BD FACSAria III cell sorter. Three independent experiments were performed in triplicate.

To detect DNA breaks in apoptotic cells *in vitro*, we used the DeadEnd Fluorometric TUNEL System (Promega) according to the manufacturer’s protocol. In brief, cells were transfected with each miR using CA using the Nunc Lab-Tek II Chamber Slide System (Fisher Scientific, Tokyo, Japan). Slides were washed 48 h post-transfection and fixed in 4% formaldehyde in PBS for 25 min at 4°C. Slides were washed twice in PBS and permeabilized in 0.2% Triton X-100 in PBS for 5 min. Slides were then washed in PBS, equilibrated in Equilibration Buffer for 7 min at room temperature, and labeled with TdT reaction mix for 60 min at 37°C in a humidified chamber to avoid light exposure. Slides were immersed in 2X SSC for 15 min to stop the reaction. Nuclei were counterstained using with ProLong Gold Antifade Reagent with DAPI. Apoptotic cells were detected by a Keyence BZ-8000 microscope.

To detect apoptotic cells *in vivo*, a TUNEL assay was performed according to the manufacturer’s protocol. Briefly, slides were de-paraffinized in Hemo-De, rehydrated in a graded ethanol series, immersed in 0.85% NaCl, and fixed in 4% formaldehyde. Cells were permeabilized with 20 μg/ml Proteinase K solution for 10 min at room temperature. Slides were equilibrated with Equilibration Buffer for 10 min at room temperature and labeled using TdT reaction mix for 60 min at 37°C in a humidified chamber. Slides were immersed in 2X SSC for 15 min to stop the reaction. Nuclei were counterstained using ProLong Gold Antifade Reagent with DAPI. Apoptotic cells were detected by a Keyence BZ-8000 microscope.

## Results

### Endogenous expression of miR-302s and miR-369s in primary tumors and cancer cell lines of colon

**Fig 1:**
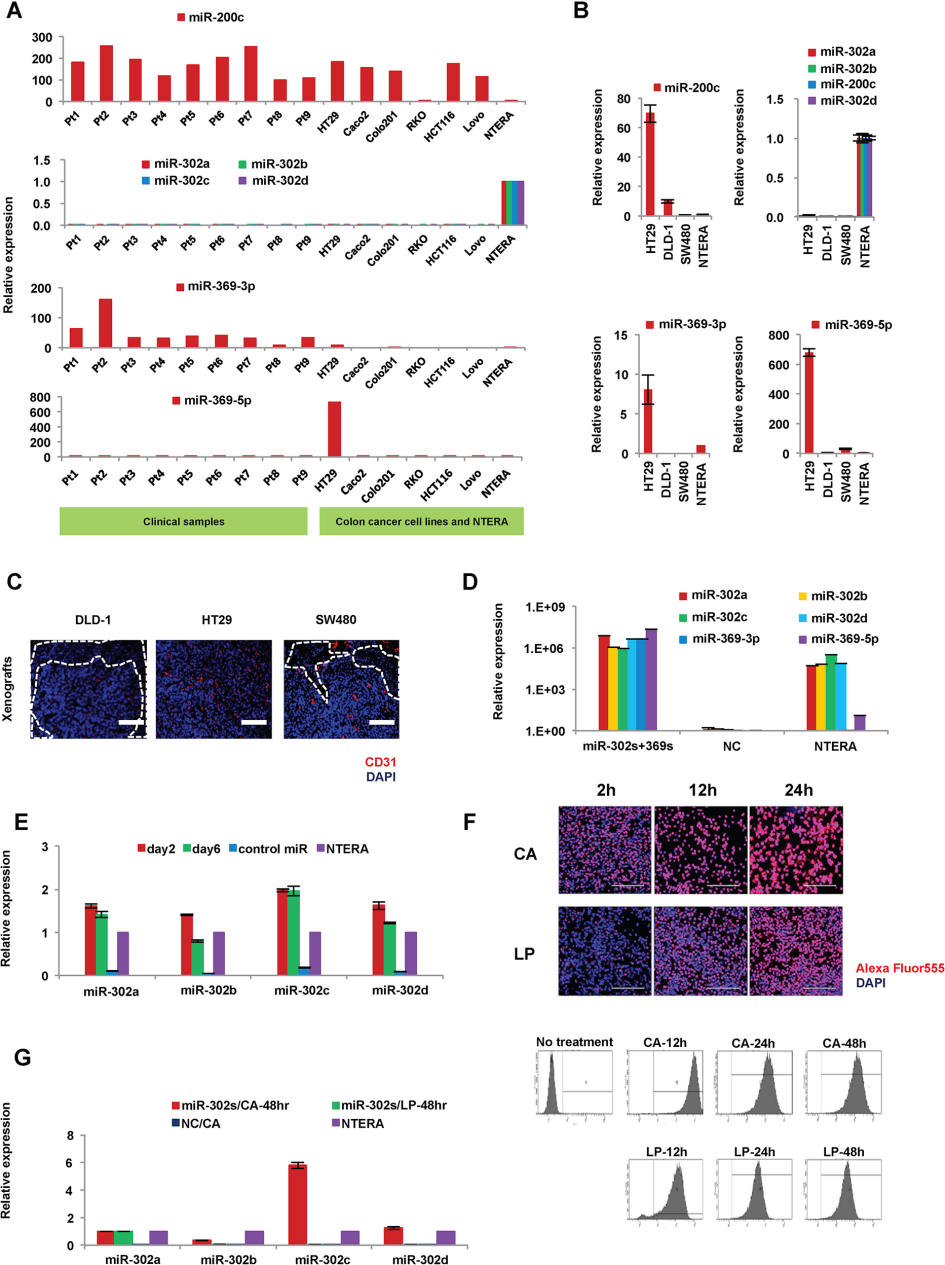
Expression of miRs in primary tumors and colon cancer cell lines. A) Relative expression of endogenous miR-302s (miR-302a,-b,-c, and-d), miR-369s (miR-369-3p and -5p), and miR-200c in primary tumors and colon cancer cell lines. The human teratocarcinoma cell line NTERA was used as a control. B) miRs expression in HT29, DLD-1 and SW480 cells (n = 3). C) Immunofluorescence analysis of CD31 expression in xenografts from HT29, DLD-1 and SW480 cells. HT29 xenografts exhibited many CD31-positive areas with little necrosis. Scale bars, 200 μm. D) Relative expression of miR-302s and miR-369s in HT29 cells 24 h post-transfection of 30 nM miR-302s plus miR-369s (n = 3). NC, negative control. E) Relative expression of miR-302s in HT29 cells at days 2 and 6 after transfection of miR-302s (n = 3). NC, negative control. F) To evaluate transfection efficiency, a fluorescently labeled dsRNA oligomer was transfected with carbonate apatite (CA) or lipofection (LP) into DLD-1 cells. Transfection efficiency was assessed by fluorescent microscopy and flow cytometry after a single transfection, as indicated. G) Relative expression of miR-302s in HT29 cells 48 h post-transfection by CA or LP (n = 3).

Previous studies indicated that miR-302a,-b,-c, and-d (miR-302s), miR369-3p, -5p (miR-369s) and miR-200c could elicit cellular reprogramming in normal somatic cells [13]. Here, we palneed to reprogram cancer cells using these miRs. We started by investigating the endogenous expression of these miRs in colorectal cancer cell lines and clinical samples of colon cancer (Fig 1A and 1B). Expression of miR-302s in colon cancer was very low or undetectable compared to that in human teratoma NTERA cells, which reportedly express ESC-specific genes and are used as control cells to evaluate ESC-like gene expression in many studies [2, 3, 5, 11]. In contrast, miR-200c expression was high in nine primary tumors examined and many cell lines, such as HT29. Expression levels of miR-302s and miR-369s were lower compared to that of miR-200c, suggesting that miR-200c expression is already high in many colon cancer cells and may be refractory to exogenous overexpression. Next, we transfected only miR-302s or miR-369s in colon cancer cells. To optimize *in vivo* experiments, which mimic primary tumors, we studied the immunofluorescent staining patterns of CD31, a vascular endothelial marker, using DAPI to counterstain nuclei, in xenografts of HT29, DLD-1 and SW480 cells (Fig 1C). HT29 xenografts had the most abundant vascularization, was uniformly distributed over the whole tumor, and the least amount of necrosis. Further cancer reprogramming analysis by miR-302s and miR-369s was performed mainly with HT29 cells and other supplementary cell lines.

### Exogenous introduction of miR-302s and miR-369s elicits cellular reprogramming

We transfected miR-302s plus miR-369s into colon cancer cell lines using CA to achieve miR expression levels similar to those of NTERA cells, which were used as a positive control (Fig 1D) [13]. We confirmed that miR-302s were effectively transfected (Fig 1E). Transfection efficiency using CA was higher than that of LP under our conditions (Fig 1F and 1G). Therefore, the CA method was used in all subsequent experiments.

**Fig 2:**
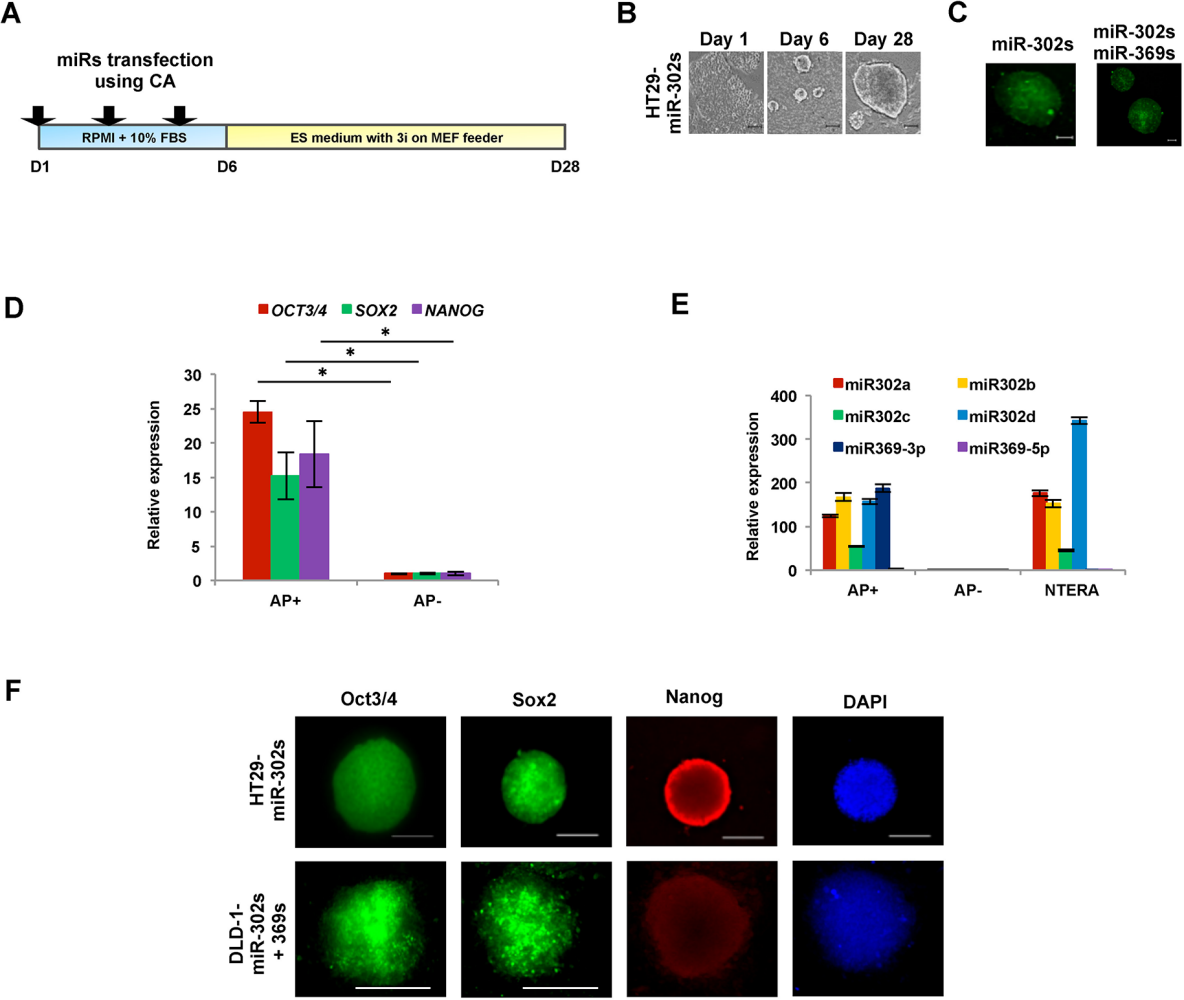
Characteristics of colon cancer cells upon reprogramming induced by miR-302s or miR-302s plus miR-369s. A) Scheme of cancer reprogramming methods by transfection of miR-302s or miR-302s plus miR-369s. B) miR-302s-induced morphological changes in HT29 cells at days 1, 6, and 28. Reprogrammed HT29 have an embryonic stem cells-like appearance. Scale bars, 100 μm. C) Cells stained using alkaline phosphatase (AP) live stain. Scale bars, 100 μm. D) Relative expression of *OCT3/4*, *SOX2* and *NANOG* compared to AP^−^ cells by qRT-PCR (n = 3). E) Relative expression of miR-302s and miR-369s in AP^+^, AP^−^, and NTERA cells. Mean expression of each miR was compared to that in NTERA cells (n = 3). F) Immunofluorescence of pluripotent stem cell markers Oct3/4, Sox2, and Nanog. Nuclei were stained with DAPI. Scale bars, 100 μm (original magnification, ×200).

To determine whether cancer cells can be reprogrammed using miRs, we examined the effect of the introduction of exogenous miR-302s with and without miR-369s three times during transfection period. On day 6, the cells were gently trypsinized, transferred onto MEF feeder cells, and cultured for additional 21 days in ES medium with three chemical inhibitors (3i; 0.5 μM A83-01, an ALK4/5/7 receptor inhibitor; 3 μM CHIR99021, a GSK-3β inhibitor; and 0.5 μM PD0325901, a MEK inhibitor) (Fig 2A) [5,13,18]. Round colonies were induced upon transfection of HT29 with miR-302s. These colonies were similar to those of ESCs/iPSCs and apparently different from those of the parent cells (Fig 2B). ESCs/iPSCs are known to be alkaline phosphatase-positive, so we stained the cells using the AP live stain (Fig 2C) to identify and pick colonies that consisted of truly reprogrammed cells [19]. Among the cells forming ES-like colonies, AP^+^ but not AP^-^ cells showed an increase the expression levels of OCT3/4, SOX2 and NANOG (Fig 2D) and miR-302s (Fig 2E). Cells transfected with miR-302s plus miR-369s or miR-302s alone were AP^+^ in culture after 28 days of reprogramming. These reprogrammed cells expressed pluripotent marker proteins such as Oct3/4, Sox2, Nanog, and TRA-1-60 (Fig 2F).

**Fig 3:**
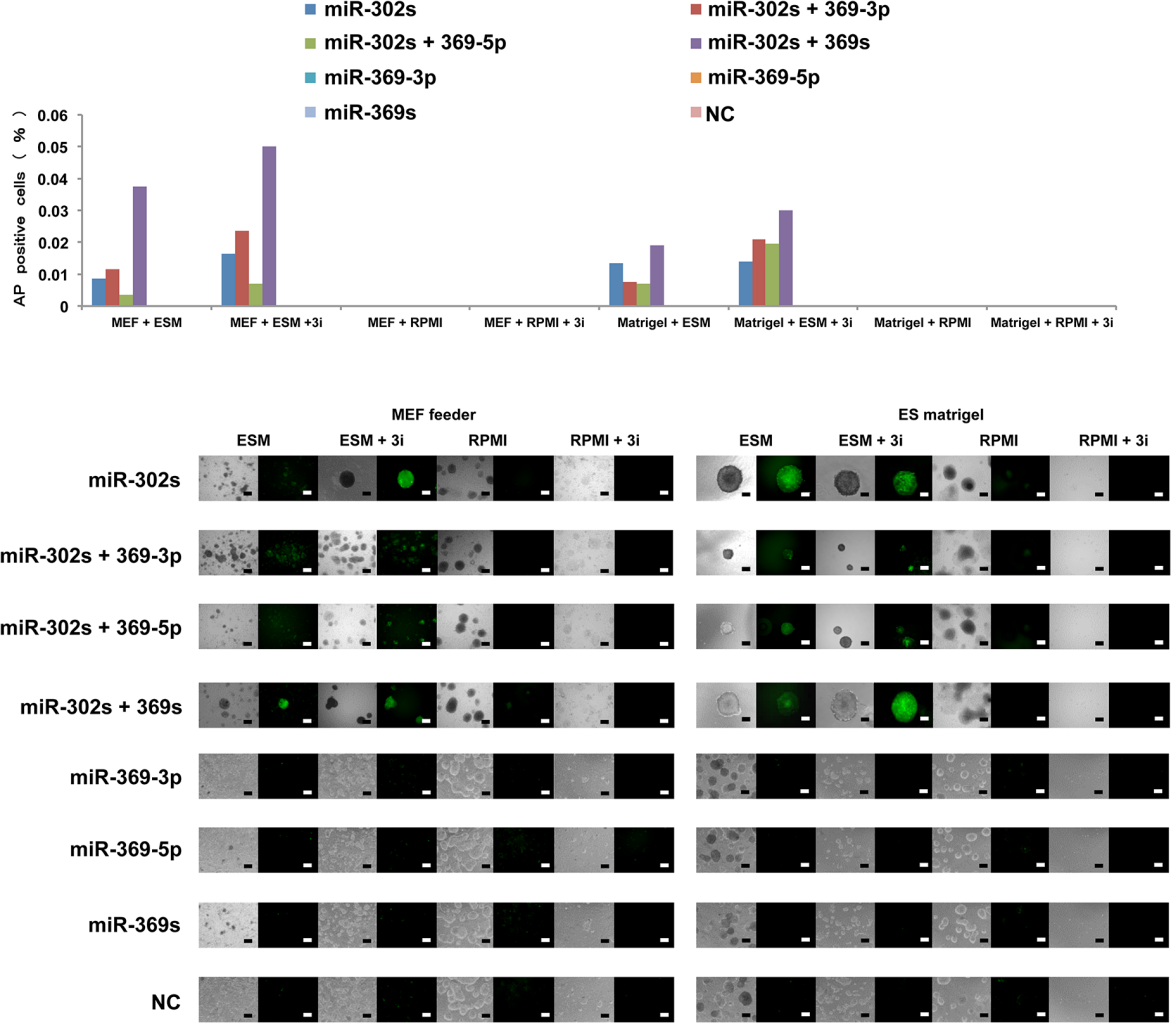
Cancer cell reprogramming conditions. Cells positive for the live stain alkaline phosphatase (AP) cells were counted as indicators of cancer reprogramming. HT29 cells were transfected with miRs as indicated and grown in culture under several conditions (culture medium, MEF feeder cells, and inhibitors). On day 21, AP staining was performed to evaluate reprogramming efficiency. Representative images are shown. Scale bars, 200 μm.

The present study indicates that AP imaging is a suitable method to analyzed not only somatic cellular reprogramming but also cancer cell reprogramming [5]. The present study also shows that cells transfected with miR-302s plus miR-369s and cultured on MEF feeder cells in ES-certified medium containing 3i exhibit a marked increase in AP^+^ ES-like colonies compared with those transfected with only miR-302s. In the absence of miRs or culturing in normal media, there is a decrease in the appearance of AP^+^ colonies (Fig 3). The data suggest that miR-302s play an important role in reprogramming cancer cells in collaboration with miR-369s. This may be facilitated by culturing in the presence of 3i on MEF feeder cells in ES-certified medium. We were therefore able to optimize the reprogramming conditions for cancer cells by utilizing synthesized compounds.

### Reprogramming cell differentiation of colon cancer

**Fig 4:**
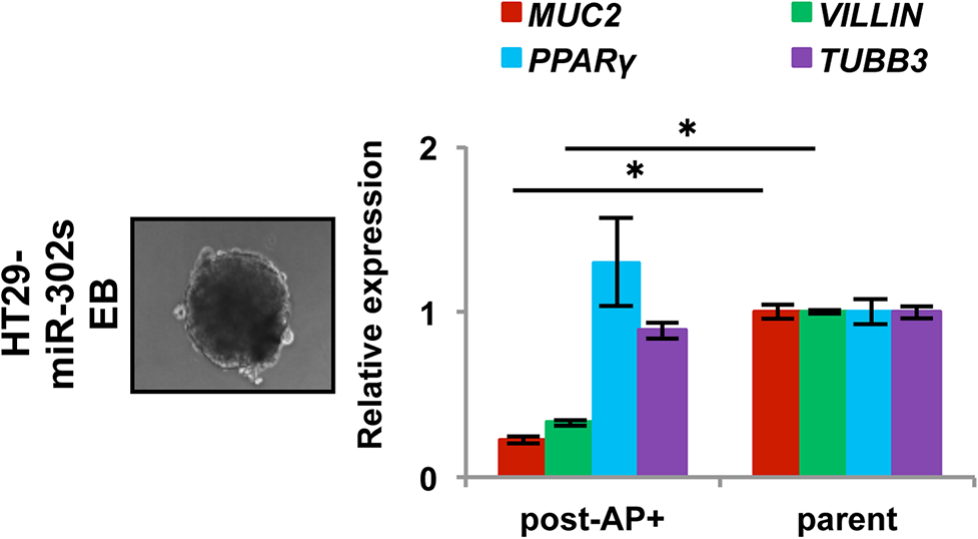
Differentiation experiment with reprogrammed cancer cells. Embryoid body formation by HT29 cells induced by miR-302s. Scale bars, 100 μm. qRT-PCR of the expression of differentiation markers, such as MUC2 and VILLIN (endodermal), PPARγ (mesodermal), and TUBB3 (ectodermal) in spontaneously differentiated AP^+^ HT29 cells.

Given that cellular reprogramming contributes cell pluripotency, we investigated whether miR-induced AP^+^ cancer cells are pluripotent. We examined EB formation in floating cultures for 4–5 days, after which the EBs were cultured on 0.1% gelatin-coated dishes for an additional 14 days to induce differentiation *in vitro*. qRT-PCR showed that expression of endodermal marker genes, such as VILLIN and MUC2, decreased, whereas expression of ectodermal marker gene TUBB3 and mesodermal marker gene PPARY did not decrease. This suggests that reprogrammed AP^+^ cells have altered expression of differentiation markers in colon cancer cells, which are largely refractory to the induction of differentiation (Fig 4).

### Epigenetic reprogramming of colon cancer cells

**Fig 5:**
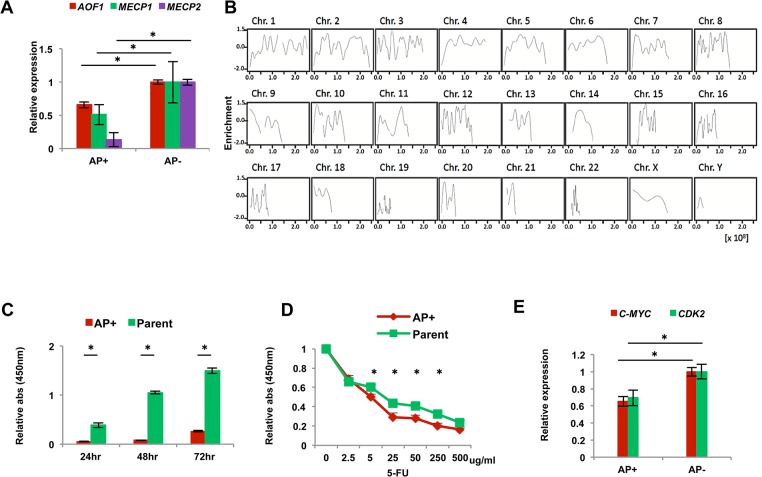
Phenotype of reprogrammed AP^+^ and AP^-^ cancer cells. A) Relative qRT-PCR expression of epigenetic regulators AOF1, MECP1 and MECP2 in AP^+^ cells compared with AP^−^ cells (n = 3). B) Demethylation data was obtained from miR-302s-transfected DLD-1 cells by immunoprecipitation and sequence analysis. Data indicate the fold of enrichment of methylation, given as a logarithmic ratio for each chromosome. Negative values indicate demethylation events upon miR-302s transfection compared to transfection with the NC. Each solid line is smoothly joined all over the chromosome. C) Cell proliferation assay with AP^+^ and parent cells (n = 12). Data are shown in comparison with the parental cells at 24, 48 and 72 h. D) 5-fluorouracil sensitivity assay of AP^+^ and parent cells (n = 4). E) Relative qRT-PCR expression of the cell cycle-related genes CDK2 and C-MYC compared with AP^−^ cells (n = 3). Asterisk denotes a p-value in the Student’s t-test of less than 0.05 (mean ± s.e.m.).

Previous reports showed that miR-302s lead to global DNA demethylation and upregulate NANOG, OCT3/4 and SOX2 via a signaling loop between miR-302s and regulatory enzymes involved in epigenetics, such as AOF1/AOF2, MECP1 and MECP2 [5,20,21]. Therefore, we compared the expression levels of those epigenetic enzyme genes between AP^+^ and AP^−^ reprogrammed cancer cells. qRT-PCR showed that AP^+^ cells expressed significantly lower levels of AOF1, MECP1, and MECP2 compared to AP^−^ cells (Fig 5A). miR-302s control the level of DNA demethylase, and we suspect that global demethylation is induced by miR-302s transfection. Therefore, we performed immunoprecipitation using an anti-DNA methylation binding protein antibody with miR-302s-transfected DLD-1 colon cancer cells. Precipitated DNA was subjected to next-generation, high-throughput DNA sequencing. Data were consistent with the idea that miR-302 induces global DNA demethylation, on the DNA sequence level on each chromosome, compared to the mock experiment (Fig 5B). This result suggests that epigenetic reprogramming events occur on the genome-wide level in AP^+^ reprogrammed cancer cells. Moreover, sequence level resolution indicated that several critical TSG loci were demethylated after miR-302s introduction, compared with mock control. This could play a role in the reactivation of the tumor suppression mechanism in malignant colon cancer cells (representative data shown in S1 Fig). Given that the reactivation of several TSGs supposedly involved in the inhibition of cancer cell growth and the sensitization of cancer cells to chemotherapeutic agents, we studied reprogrammed colon cancer cells *in vitro*. The data indicate that reprogrammed AP^+^ cancer cells have reduced cell proliferation (Fig 5C) and a sensitized phenotype to exposure to 5-FU, chemotherapeutic agent, in culture (Fig 5D). This is consistent with previous reports in other tumors [1,5,18]. The oncogene c-MYC is often involved in somatic cellular reprogramming and is an inevitable problem in tumorigenesis [22]. The present study indicates that the change of malignant phenotype might be due to decreased levels of c-MYC and CDK2 in AP^+^ cells (Fig 5E), suggesting a potential therapeutic application.

### miR-302s and miR-369s induce mesenchymal-to-epithelial transition phenotypes

**Fig 6:**
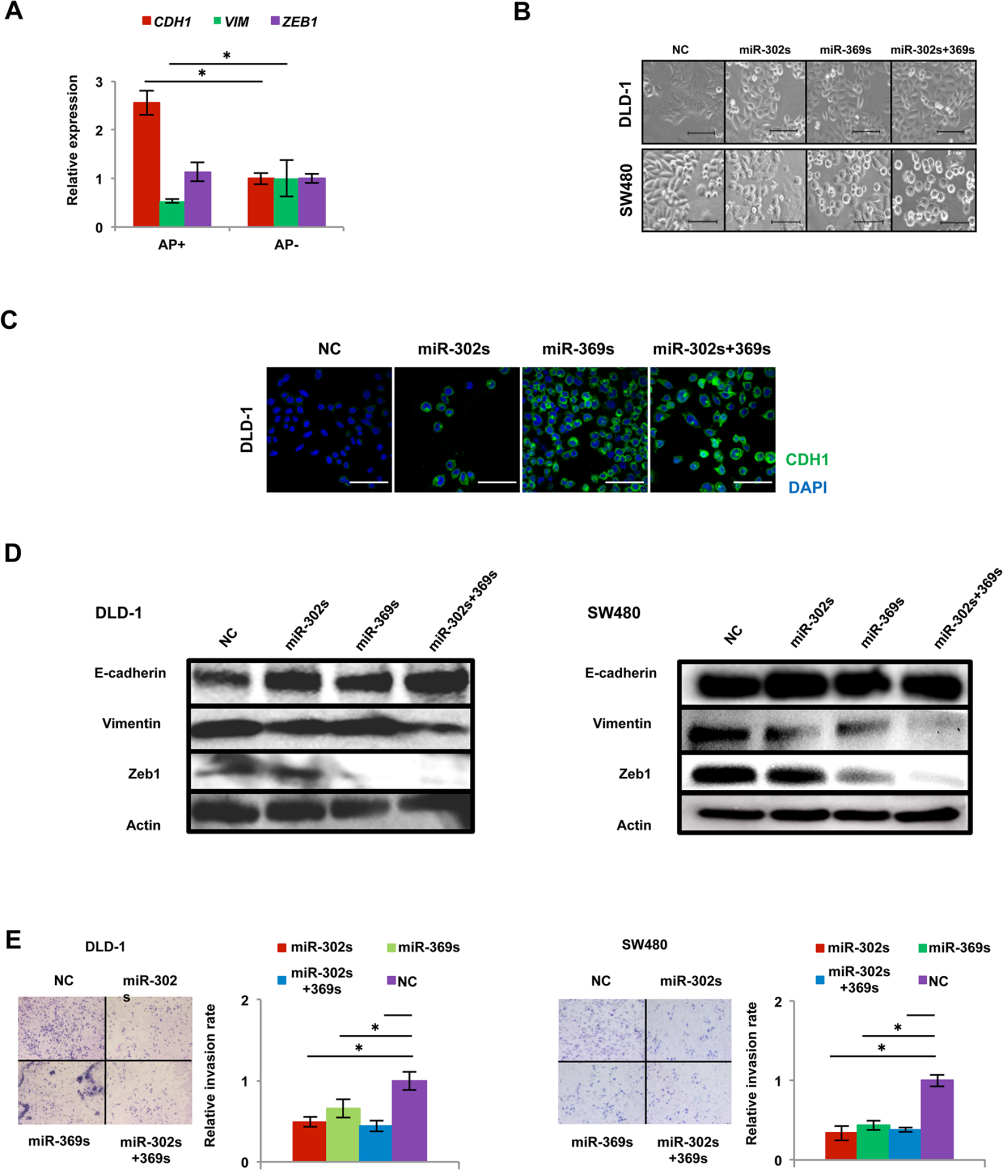
miR-302s plus miR-369s induce the mesenchymal-to-epithelial transition phenotype. A) Relative qRT-PCR expression of MET-related genes CDH1, VIMENTIN (VIM) and ZEB1 compared with AP^−^ cells (n = 3). B) Change in morphology of DLD-1 and SW480 cells after transfection of each miR. Scale bars, 100 μm. C) Immunofluorescence analysis of MET-related protein CDH1 in DLD-1 cells 48 h after each miR transfection. Nuclei were counterstained by DAPI. Scale bars, 100 μm. D) Immunoblotting for MET-related proteins E-cadherin, Vimentin, and Zeb1. Actin was used as a loading control. Left, DLD-1 cells; right, SW480 cells. E) Matrigel invasion assay of DLD-1 cells and SW480 cells. Scale bars, 100 μm. Relative invasion rates were determined by counting the number of cells that invaded through Matrigel compared to that in the mock control miR. Data are obtained from three independent experiments. Asterisk denotes a p-value in the Student t-test of less than 0.05 (mean ± s.e.m.).

Somatic cellular reprogramming largely requires the mesenchymal-to-epithelial transition (MET), orchestrated by the suppression of pro-EMT, linked to aggressive cancer stem cell phenotypes [23,24]. AP^+^ cells showed an increase in CDH1 expression and a decrease in VIMENTIN expression compared with AP^-^ cells (Fig 6A). Colon cancer cells transfected these miRs underwent a phenotype change from spindle-shaped to round-shaped in colon cancer cells (Fig 6B), consistent with an increase in CDH1 protein levels (Fig 6C and 6D). Interestingly, protein expression of Zeb1, a mesenchymal regulator, was also decreased in cells transfected with miR-369s (Fig 6D). An invasion assay using Matrigel-coated membranes demonstrated that transfection with miR-302s plus miR-369s resulted in a significant decrease in cancer cell invasion (Fig 6E). These results suggest that miR-302s plus miR-369s accelerate MET in colon cancer cells.

### Cancer reprogramming inhibits the Cdk–Cyclin axis

**Fig 7:**
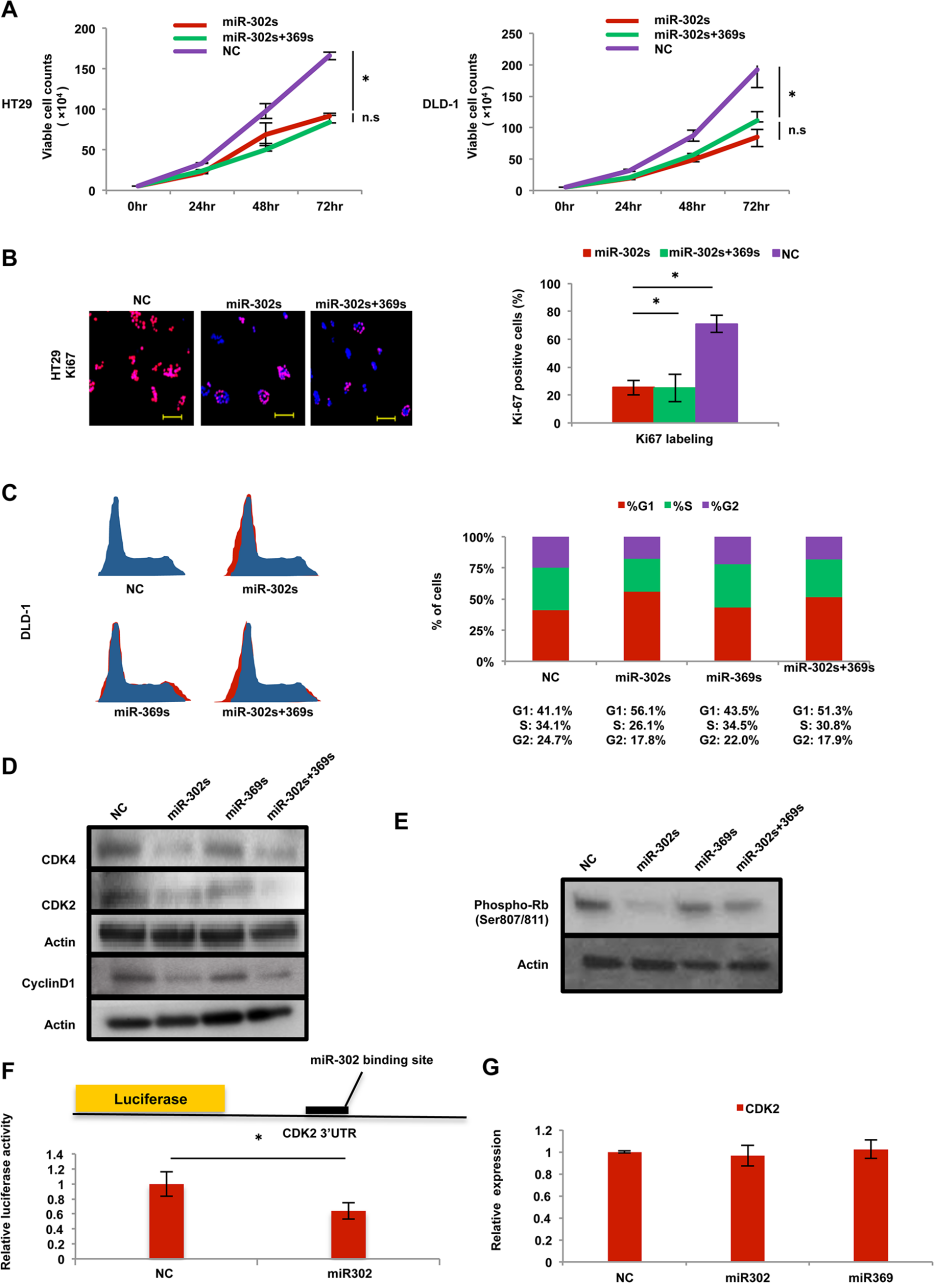
Effect on cell cycle and proliferation. A) Cell growth assay of HT29 and DLD-1 cells transfected with miR-302s, miR-302s plus miR-369s, or NC miR. Viable cells were counted 24, 48 and 72 h after transfection (n = 3). B) Immunofluorescence staining of proliferating cells by Ki67. HT29 cells were transfected with miR-302s, miR-302s plus miR-369s, or negative control (NC) miR. Positively stained-cells were counted from three random fields in three independent experiments. Scale bars, 100 μm (original magnification, ×200). C) Cell cycle analysis of DLD-1 cells transfected with miR-302s, miR-302s plus miR-369s, or NC miR. Cells were stained by propidium iodide. Left, representative image; right, median cell populations (%) in three cell cycle phases (G1, S and G2) from three independent experiments. D) Immunoblotting of CDK4, CDK2, and CyclinD1 in DLD-1 cells transfected with miR-302s, miR-369s, miR-302s plus miR-369s, or NC miR. Actin was used as a loading control. E) Immunoblotting with anti-phosphorylated Rb (Ser807/811) antibody in DLD-1 cells transfected with miR-302s, miR-369s, miR-302s plus miR-369s, or NC miR. Actin was used as a loading control. F) Schematic of CDK2-3’-UTR-containing reporter constructs. A luciferase reporter assay with DLD-1 cells transfected with a luciferase-CDK2 3’UTR vector and miR-302s was performed. NC, negative control. Data were normalized by empty vector co-transfection and were obtained from three independent experiments. G) qRT-PCR of CDK2 expression in DLD-1 cells 48 post-transfection with miR-302s, miR-369s, or NC miR (n = 3). Relative ratios are shown. Asterisk denotes a p-value in the Student t-test of less than 0.05 (mean ± s.e.m).

We studied further the miR-induced effect on cell proliferation. Introduction of miR-302s and miR-302s plus miR-369s resulted in the inhibition of cell proliferation in colon cancer cells (Fig 7A). Immunocytochemistry showed that transfection of miR-302s resulted in the reduction of Ki67-positive cells (Fig 7B). A cell cycle study of PI staining using flow cytometry showed that the percentage of cells in G1-phase was relatively increased compared with the NC (Fig 7C), suggesting the miR-302s inhibits entry into the S-phase of the cell cycle. Immunoblot analysis also showed that CyclinD1, Cdk2 and Cdk4 proteins were decreased in miR-302s-transfected cells (Fig 7D), These results suggest that Cyclin/Cdk proteins are involved in miR-dependent cell cycle inhibition. To address this hypothesis further, we studied the phosphorylation of the Rb protein, a target of Cyclin/Cdk. Immunoblotting showed that miR-302s markedly inhibited Rb phosphorylation (Fig 7E). To determine whether miR-302s directly controls the 3’UTR of the CDK2 gene, we performed a luciferase reporter assay. The result showed that relative firefly luciferase activity of reporter plasmids containing the CDK2 3’ UTR binding site was significantly inhibited in colon cancer cells transfected with miR-302s (Fig 7F), indicating that miRs inhibits Cdk2 protein (Fig 7D), but not CDK2 mRNA (Fig 7G). These data suggest that miR-mediated reprogramming modulates the Cyclin–Cdk axis, a mechanism critical in cancer cell proliferation.

### Induction of apoptosis in colon cancer cells

Since cell death was observed during the initial transfection period, we examined apoptosis using the fluorescent TUNEL assay. Data showed that transfection with the miRs induced the development of TUNEL-positive cells (A in S2 Fig). The results were confirmed by Annexin V staining, which demonstrated that miR-302s induced early and late apoptosis (B in S2 Fig). Immunoblotting showed that expression of the apoptosis-related Bcl-2-family protein Mcl-1 was decreased in miR-302s-transfected colon cancer cells (C in S2 Fig), suggesting an involvement of the mitochondrial Bcl-2-family in miR-302s-induced apoptosis.

### miR-mediated cancer reprogramming therapy *in vivo*

**Fig 8:**
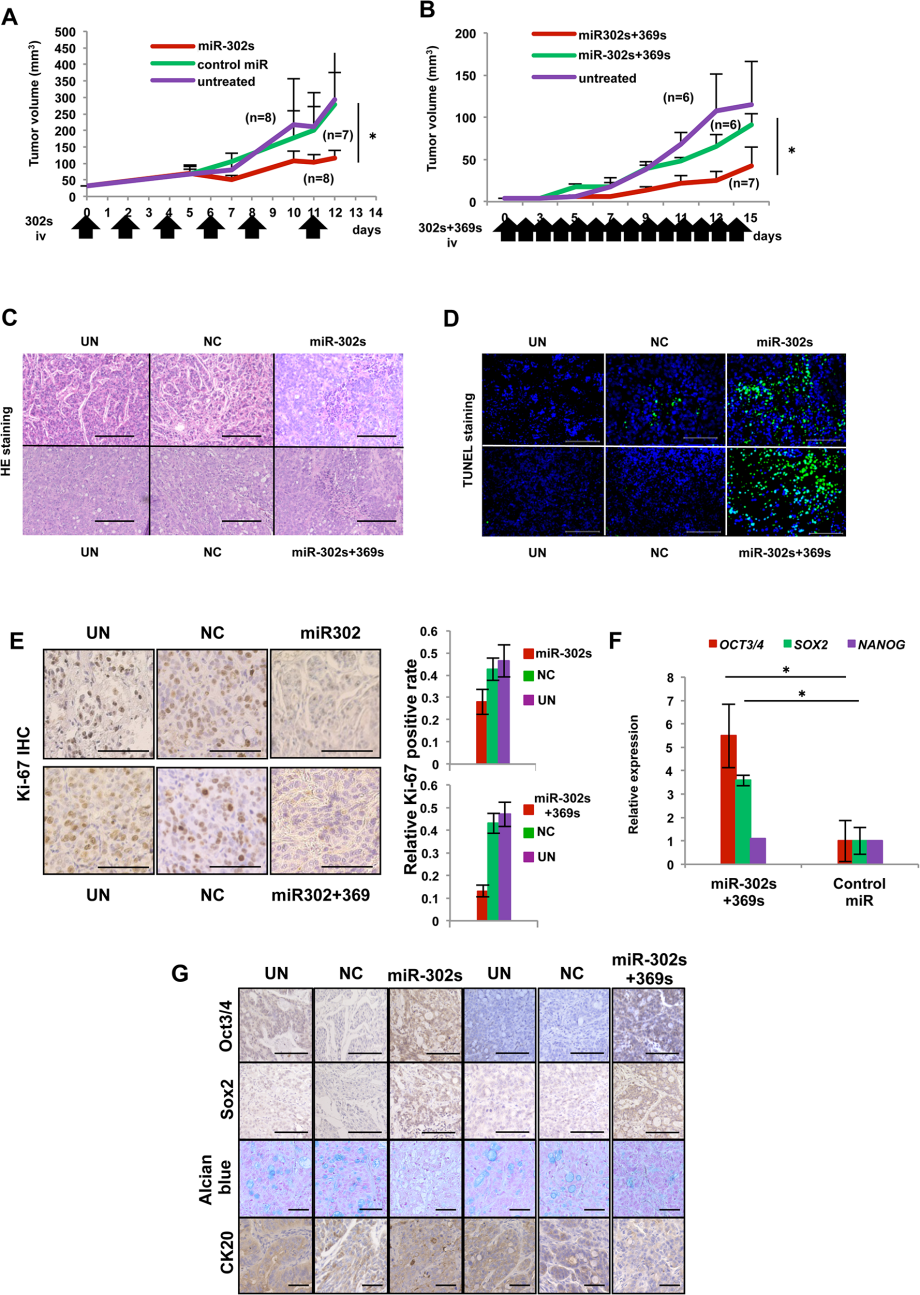
Anti-tumor effect by systemic administration of miR-302s plus miR-302s *in vivo*. A) Tumor-bearing mice were treated with systemic administration of carbonate apatite (CA)- complexed miR-302s, negative control (NC) miR, or CA only every second day, with a total of six injections. Tumor growth curves are shown. Asterisk denotes a p-value in the Mann-Whitney U-test of < 0.05. B) Tumor-bearing mice were treated with systemic administration of CA-equipped miR-302s plus miR-369s, NC miR, or CA only daily, with a total of 14 injections, similar to (A). C) Representative hematoxylin and eosin-stained images of tumor sections from the indicated experimental groups. Areas with denaturated nuclei were predominantly observed in xenografts treated with miR-302s/CA complexes and miR-302s plus miR-369s/CA complexes. Scale bars, 100 μm (magnification ×200). D) Confirmation of intratumoral apoptosis by TUNEL staining in tumor sections obtained from xenografts treated with CA-mediated miR-302s, miR-302s plus miR-369s, NC, or CA only. Scale bars, 100 μm (magnification ×200). E) Immunohistochemistry analysis of the proliferation marker Ki-67 in xenografts treated with miR-302s, miR-302s plus miR-369s, NC miR or the mock control. Representative images are shown. Scale bars, 100 μm (magnification ×200). Ki-67-positive cells were counted in three random fields from different three xenografts in each group. F) Relative expression of pluripotency-related markers OCT3/4, SOX2 and NANOG in the indicated experimental groups. Data are shown in comparison with the NC miR group. (n = 3). G) Representative immunohistochemical images of tumor sections from the indicated experiment groups. Oct3/4 and Sox2 were predominantly observed in xenografts treated with miR-302s and miR-302s plus miR-369s. Alcian blue staining showed the decrease of mucin production in these miR-treated-groups. CK20 expression was also decreased in xenografts treated with miR-302s plus miR-369s. Scale bars, 100 μm (magnification ×200).

The results *in vitro* show that miR-302s and miR-369s play a role as tumor suppressor miRs and influence cancer reprogramming, acceleration of MET, inhibition of the Cyclin/Cdk axis, suppression of cell growth, and apoptosis. Therefore, we also analyzed the effect of these miRs *in vivo*. Because miRs are unstable *in vivo*, miR-302s was administered by injection into the tail vein six times. Systemic administration of miR-302s resulted in the inhibition of tumor growth compared with the control miR and a mock experiment as controls (Fig 8A). We then studied the combination of miR-302s and miR-369s by injecting these miRs into the tail vain 14 times. Administration of miR-302s and miR-369s also resulted in the inhibition of tumor growth compared with the control miR and experimental control groups (Fig 8B). Those two experiments demonstrated that significant tumor growth inhibition occurs when mice are with miR-302s alone or miR-302s and miR-369s compared with controls, without apparent adverse effects. Histological examination of these tumor tissues showed a focal involvement of cell debris with denatured nuclei in tumors (Fig 8C). TUNEL staining confirmed DNA fragmentation, a characteristic of apoptosis (Fig 8D). The number of Ki67-positive cells was significantly decreased in miRs-treated tumors compared with NC miR tumors (Fig 8E). In terms of tumor differentiation, it was revealed that miR-302s plus miR-369s-treated tumors were strongly positive for Oct3/4 and Sox2 (Fig 8F and 8G). Alcian blue staining for goblet-like cell differentiation showed a decrease in mucus production in tumors treated with miR-302s alone or miR-302s plus miR-369ss (Fig 8G). CK20, a differentiation marker for colon adenocarcinoma, was also repressed in tumors treated with miR-302s plus miR-369ss (Fig 8G).

**Fig 9:**
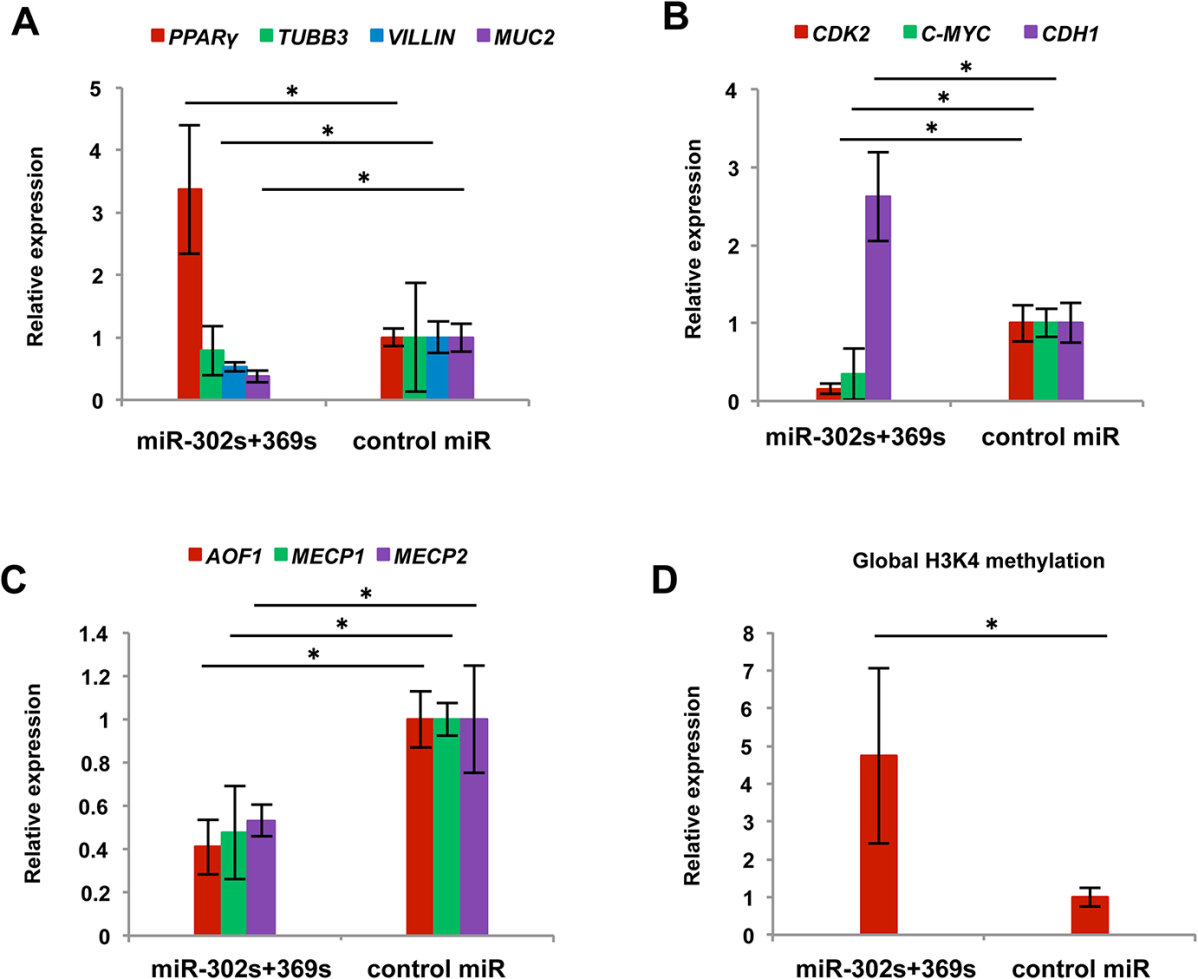
Gene expression of reprogrammed cancer cells *in vivo*. A) Relative expression of the differentiation-related genes PPARγ, TUBB3, VILLIN and MUC2 in the indicated experimental groups. Data are shown in comparison with the negative control (NC) miR group. (n = 3). B, C) Relative expression of CDK2, C-MYC and CDH1 (B) and epigenetic regulators AOF1, MECP1 and MECP2 (C) in the indicated experimental groups. Data are shown in comparison with the NC miR group. (n = 3). D) Relative global histone 3 lysine-4 methylation status of xenografts. Data are shown in comparison with the NC miR group. (n = 3). Asterisk denotes a p-value in the Student t-test < 0.05 (mean ± s.e.m.).

qRT-PCR indicated that expression of MUC2 and VILLIN, enterocyte-like cell differentiation markers associated with axial microfilaments and conserved in adenocarcinoma [25], decreased, whereas expression of PPARY increased (Fig 9A). These results showed miR-302s plus miR-369s modulate the colonic differentiation state and could elicit a shift toward another lineage *in vivo*. Moreover, we confirmed the decrease of CDK2 and c-MYC and increase of CDH1 expression at the mRNA level *in vivo*, consistent with the results *in vitro* (Fig 9B). We next studied the alteration of epigenetic regulators in tumors treated with miR-302s plus miR-369ss. Results showed that *AOF1*, *MECP1 and MECP2* expression levels were lower in miR-302s plus miR-369ss-treated tumors (Fig 9C). Therefore, we studied global H3K4 methylation. The results indicated that H3K4 methylation was increased in miR-302s plus miR-369s-treated tumors (Fig 9D) and suggested that miR-302s plus miR-369s may decrease AOF1, MECP1 and MECP2 expression, leading to global epigenetic modification [20].

**Fig 10:**
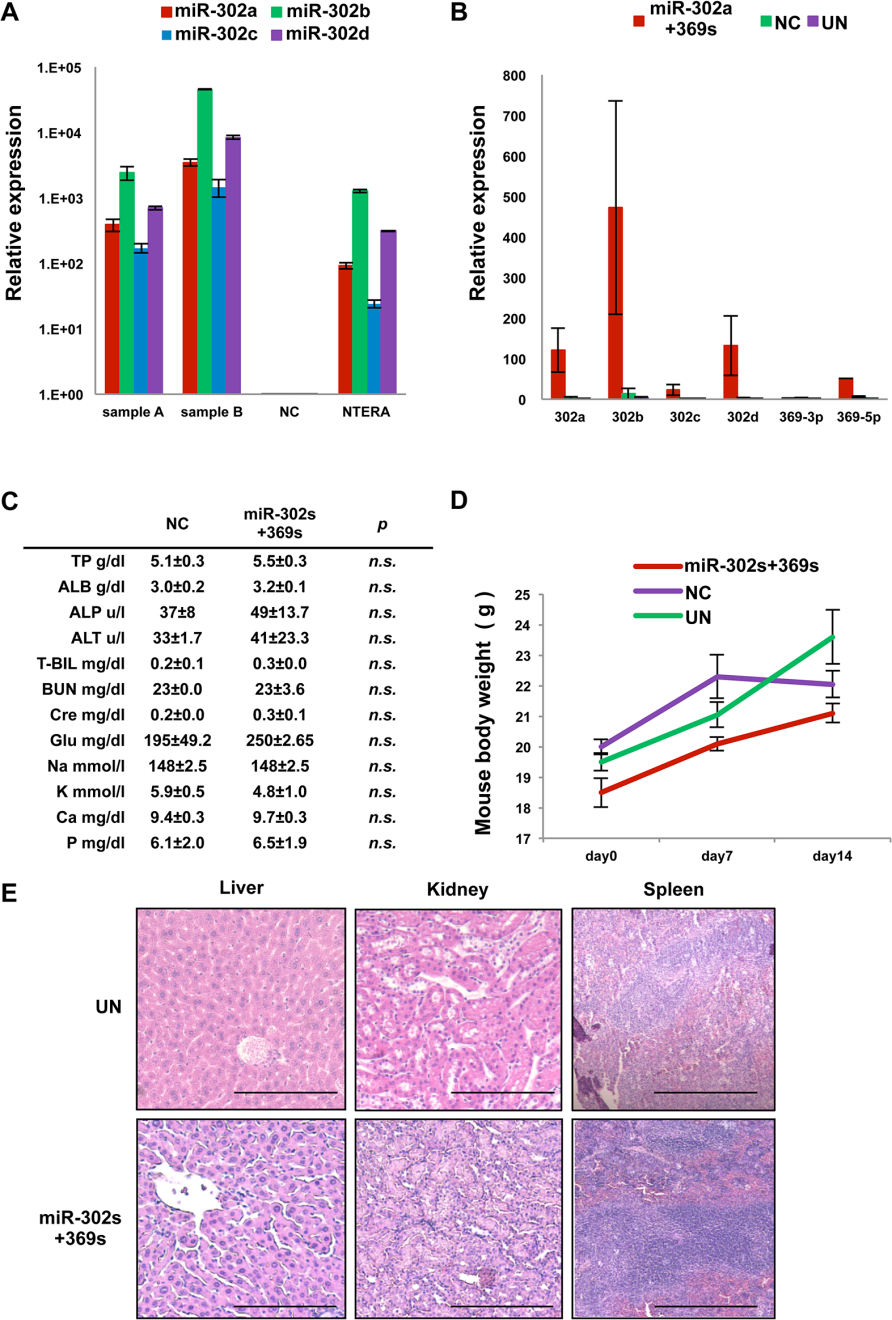
miR distribution and adverse effects of systemic administration. A) Relative expression of miR-302s in xenografts 24 h after intravenous injection with miR-302s two or three times daily. NTERA was used as a positive control for miR-302s. Mean expression of each miR was compared with that of the negative control (NC) miR group (n = 3). B) Relative expression of miR-302s and miR-369s in xenografts treated with miR-302s plus miR-369s, NC miR, or the mock control. Tumors were harvested 3 d after injection of the indicated miRs on 14 consecutive days. Mean expression was compared with that in the mock treatment (n = 3). C) Mean values of biochemical parameters in the serum of mice treated with 14 consecutive daily injections of miR-302s plus miR-369s or NC miR (n = 4). D) Monitoring of the body weight of mice treated with 14 consecutive daily injections of miR-302s plus miR-369s, NC miR, or the mock control(n = 4). E) Representative images of hematoxylin and eosin staining of the liver, kidney and spleen from mice treated with 14 consecutive daily injections of miR-302s plus miR-369s or the mock control. Scale bars, 200 μm. Asterisk denotes a p-value in the Student t-test of < 0.05 (mean ± s.e.m.).

We performed qRT-PCR to investigate whether systemic administration of miRs was distributed into the deep areas of tumors. The data indicated that systemic administration of miRs led to excellent distribution in xenografts (Fig 10A and 10B). Although systemic administrations of miR-302s plus miR-369s were repeated for 14 consecutive days, we did not detect any noticeable adverse effects in laboratory blood tests, body weight or histology of the liver, kidney and spleen (Fig 10C, 10D and 10E).

## Discussion

Epigenetic events are critical in dictating cellular differentiation and reprogramming of somatic and cancer cells [1, 2]. The present study showed that the combination of miR-302s plus miR-369s could modulate biological behaviors of colorectal cancer cells. A state-of-the-art chromatin immunoprecipitation sequencing experiment demonstrated that global epigenetic alterations such as DNA demethylation occur in miR-302s-transfected colorectal cancer cells. This may be beneficial in reactivating tumor suppressor genes, usually inactivated during the establishment of cancer through a complex series of epigenetic events [1]. We also found that administration of miRs resulted in methylation of H3K4, an active marker of chromatin that is often inactivated by abnormal epigenetic control by the p16/INK4A tumor suppressor. Reportedly, H3K4 plays a critical role in the cell cycle control of cancer stem cells, acting to slow down cycling [1]. Given the present study showed that a combination of miR-302s and miR-369s induced epigenetic modulations of histones and DNA, there may be a justification for the use of these miRs as novel therapeutic agents.

Here, we used miR-302s and miR-369s, based on expression analysis, to boost endogenously low expression with exogenously high expression. In this regard, we did not use miR-200c in this study, since the additional expression of miR-200c in endogenously miR-200c-expressing cancer cells did not indicate an apparent anti-cancer effect. One of the common features shared by these three miRs, which can induce cellular reprogramming in somatic cells [11], is the ability to inhibit the epithelial–to–mesenchymal transition (EMT). Reportedly, the miR-200 family is recognized as a master regulator of the epithelial phenotype by targeting ZEB1 and ZEB2, two important transcriptional repressors of cell adherence (E-cadherin) and polarity (CRB3 and LGL2) genes [22]. The miR-200 family has also been shown to inhibit the EMT, stimulating the epithelial phenotype and cancer cell migration by direct targeting of the E-cadherin transcriptional repressors ZEB1 and ZEB2 [23]. The miR-302s target several important molecules, such as the receptor for TGFβ (α στρονγ ινδυχερ οϕ EMT), histone H3K4 demethylase [24], and Cdk2/4/6 [12]. The miR-369s alone inhibit the EMT and stimulate the epithelial phenotype. The present study indicated that miR-369s inhibit the EMT in collaboration with miR-302s, which is compatible with a database search that indicates that miR-369s target ZEB1 (http://www.microrna.org/microrna/home.do). We hypothesize that miR-369s exert part of their function of EMT inhibition in collaboration with other miRs. Thus, similarly to iPS induction stimulated by the epithelial program [25], inhibition of the mesenchymal phenotype by these miRs may be beneficial in eradicating the metastatic potential of cancer. Eventually, the introduction of miR-302s should result in the induction of reprogramming [5] and the suppression of the malignant potential of tumors [12].

Considering cellular reprogramming, ‘the c-Myc issue’ may be important. It has been reported that c-Myc is involved in reprogramming events and inevitably activated during the course of cellular reprogramming through virus-mediated reprogramming, which can lead to carcinogenesis [22,26]. The present study indicates that a combination of miR-302s and miR-369s results in the suppression of endogenous c-Myc and inactivates tumors. Thus, miR-based reprogramming may be beneficial, given that a combination of miR-302s and miR-367 could carry out reprogramming without apparent c-Myc alterations [8,9,11,20,21]. The present study shows that introduction of the miRs resulted in sensitization to 5-FU, induction of the G0/G1-phases of the cell cycle, and induced apoptosis via the mitochondrial Bcl-2 family. An *in vivo* study indicated that miRs modulated the differentiation process, and may direct other differentiation lineages and inactivate malignant potential, as detected by differentiation markers CK20 and Alcian blue. Thus, we suspect that reprogramming therapy may overcome resistance against conventional chemotherapy by epigenetic modifications, although further studies are undoubtedly necessary. In the application of nucleotide medicine *in vivo*, a proper drug delivery system may facilitate targeting efficiency against cancer stem cells [27,28]. Chemical modifications, such as bridged nucleotides, might be beneficial in increasing the anti-cancer effects and decreasing off-target effects [27,28]. Since small RNAs are unlikely to be incorporated into DNA strands in the nucleus, a reprogramming strategy may be worth considering as a novel future treatment strategy [27,28].

## Supporting Information

S1 Fig DNA methylation of around tumor suppressor gene (± 10 Kb). Each solid lines are fitted by Gaussian function. The intensities and the deviation of function correspond to fold enrichment and the detected sequence range, respectively.
(TIF)

S2 Fig Apoptotic induction. A, Fluorescent TUNEL staining was performed to detect apoptotic HT29 cells transfected with miR302, miR302 plus miR-369s, or negative control (NC) miR. Apoptotic cells are indicated by an arrow. B, Propidium iodide and Annexin V-FITC staining was performed in DLD-1 cells 60 h post-transfection with miR302, miR-369s, miR302 plus miR-369s, or NC miR. Apoptotic cells were measured by flow cytometry. Early (Annexin-positive only) and late (both Annexin and PI-positive) apoptotic cells were detected. Three independent experiments were performed. C, Immunoblotting of the apoptosis-related proteins Bak, Bid, Bcl-xl, Bcl2, Mcl1, Caspase-8, and Caspase-3 in HT29 and DLD-1 cells transfected with miR302, miR-369s, miR302 plus miR-369s, or NC miR. Actin was used as a loading control. Asterisk denotes a p-value in the Student t-test of < 0.05 (mean ± s.e.m.).
(TIF)

S1 Table Sequence of mature miRNAs. (TIFF)

S2 Table Patients characteristics of clinical samples. Stage was according to TNM classification (UICC 7th) Abbreviations; CEA.carcinoembryonic antigen, CA19-9.carbohydrate antigen 19–9, RS. Rectosigmoid, Ra. Upper rectum, Rb.Lower rectum A. Ascending, D. Descending, S.Sigmoid, wel. well differentiated adenocarcinoma, mod. moderately differentiated adenocarcinoma.
(TIFF)
