# Supplementary figures and images for: MicroRNAs Induce Epigenetic Reprogramming and Suppress Malignant Phenotypes of Human Colon Cancer Cells
Source: PLoS One. 2015 May 13;10(5):e0127119. doi: 10.1371/journal.pone.0127119 (PMC4430240; doi:10.1371/journal.pone.0127119)

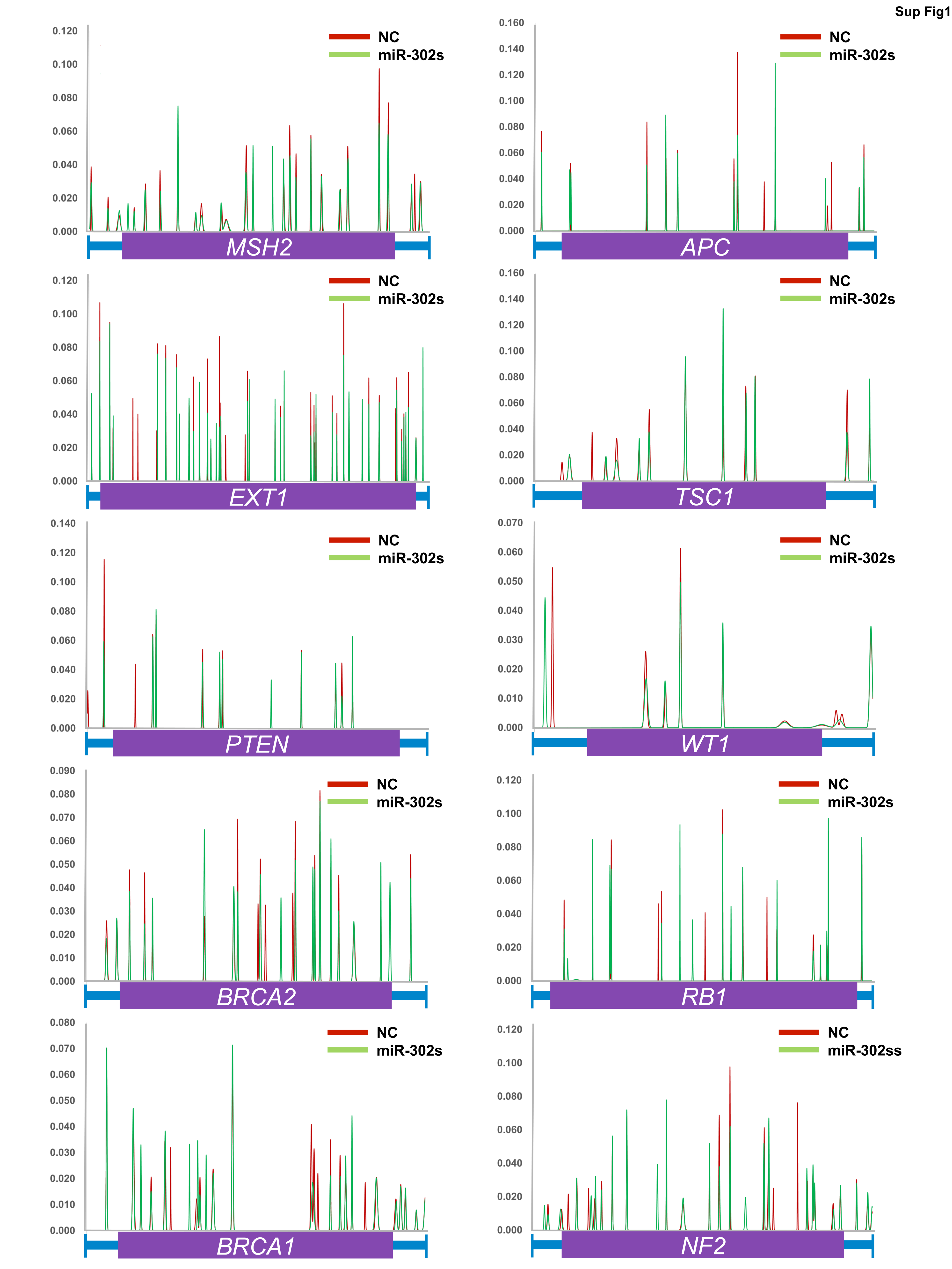

Supplement: S1 Fig — Each solid lines are fitted by Gaussian function. The intensities and the deviation of function correspond to fold enrichment and the detected sequence range, respectively. (TIF) [file pone.0127119.s001.tif]

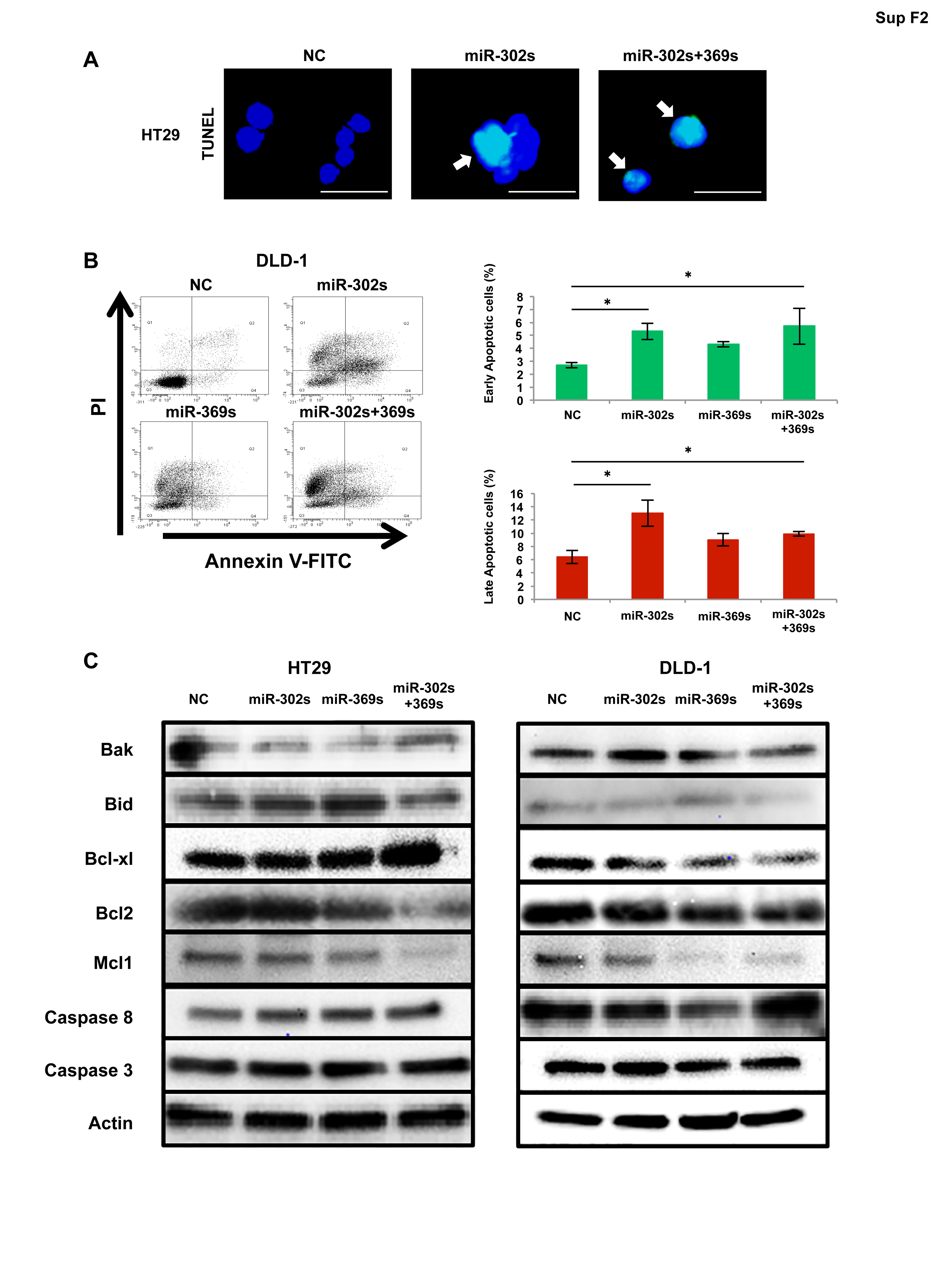

Supplement: S2 Fig — A, Fluorescent TUNEL staining was performed to detect apoptotic HT29 cells transfected with miR302, miR302 plus miR-369s, or negative control (NC) miR. Apoptotic cells are indicated by an arrow. B, Propidium iodide and Annexin V-FITC staining was performed in DLD-1 cells 60 h post-transfection with miR302, miR-369s, miR302 plus miR-369s, or NC miR. Apoptotic cells were measured by flow cytometry. Early (Annexin-positive only) and late (both Annexin and PI-positive) apoptotic cells were detected. Three independent experiments were performed. C, Immunoblotting of the apoptosis-related proteins Bak, Bid, Bcl-xl, Bcl2, Mcl1, Caspase-8, and Caspase-3 in HT29 and DLD-1 cells transfected with miR302, miR-369s, miR302 plus miR-369s, or NC miR. Actin was used as a loading control. Asterisk denotes a p-value in the Student t-test of < 0.05 (mean ± s.e.m.). (TIF) [file pone.0127119.s002.tif]

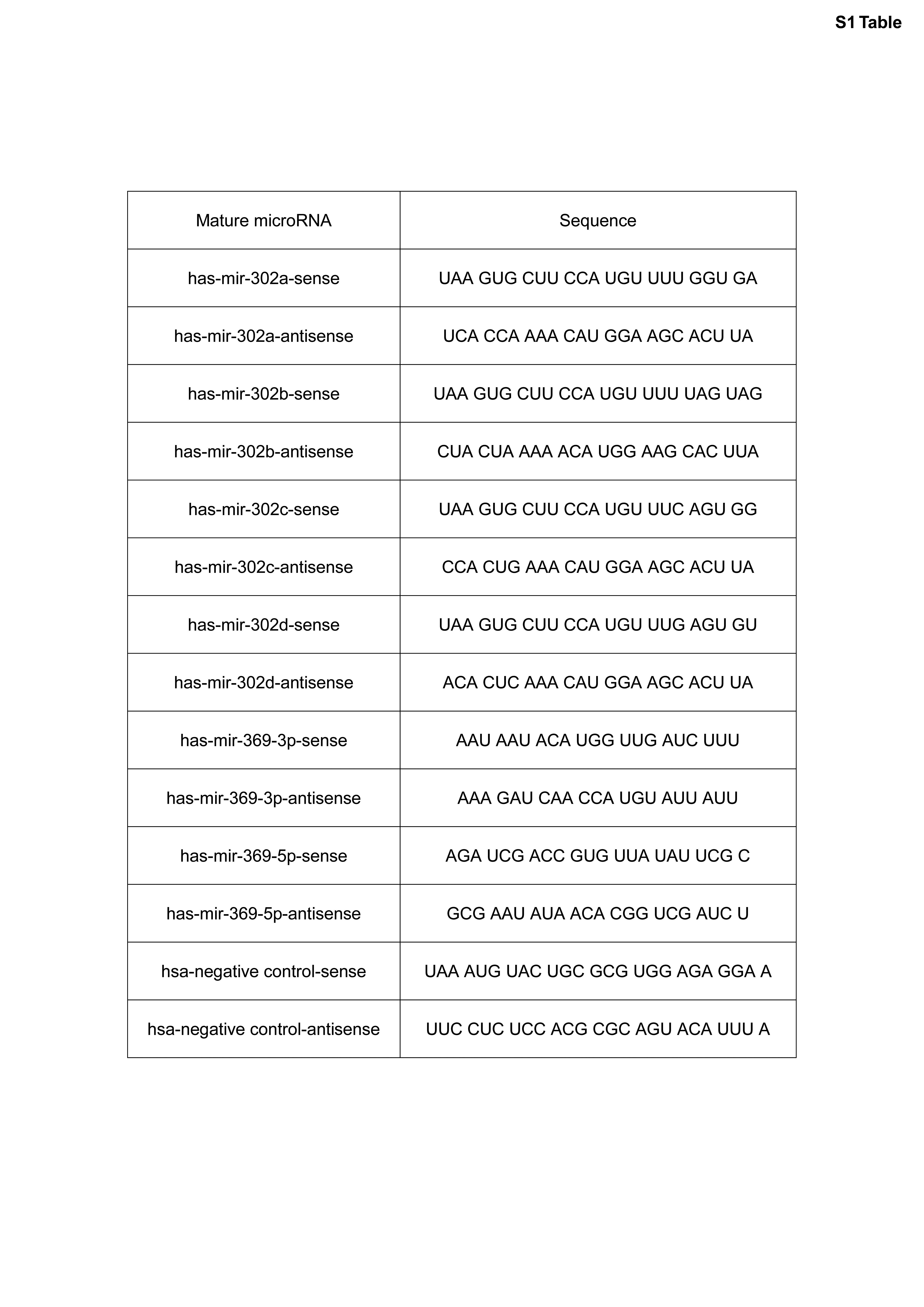

Supplement: S1 Table — (TIFF) [file pone.0127119.s003.tiff]

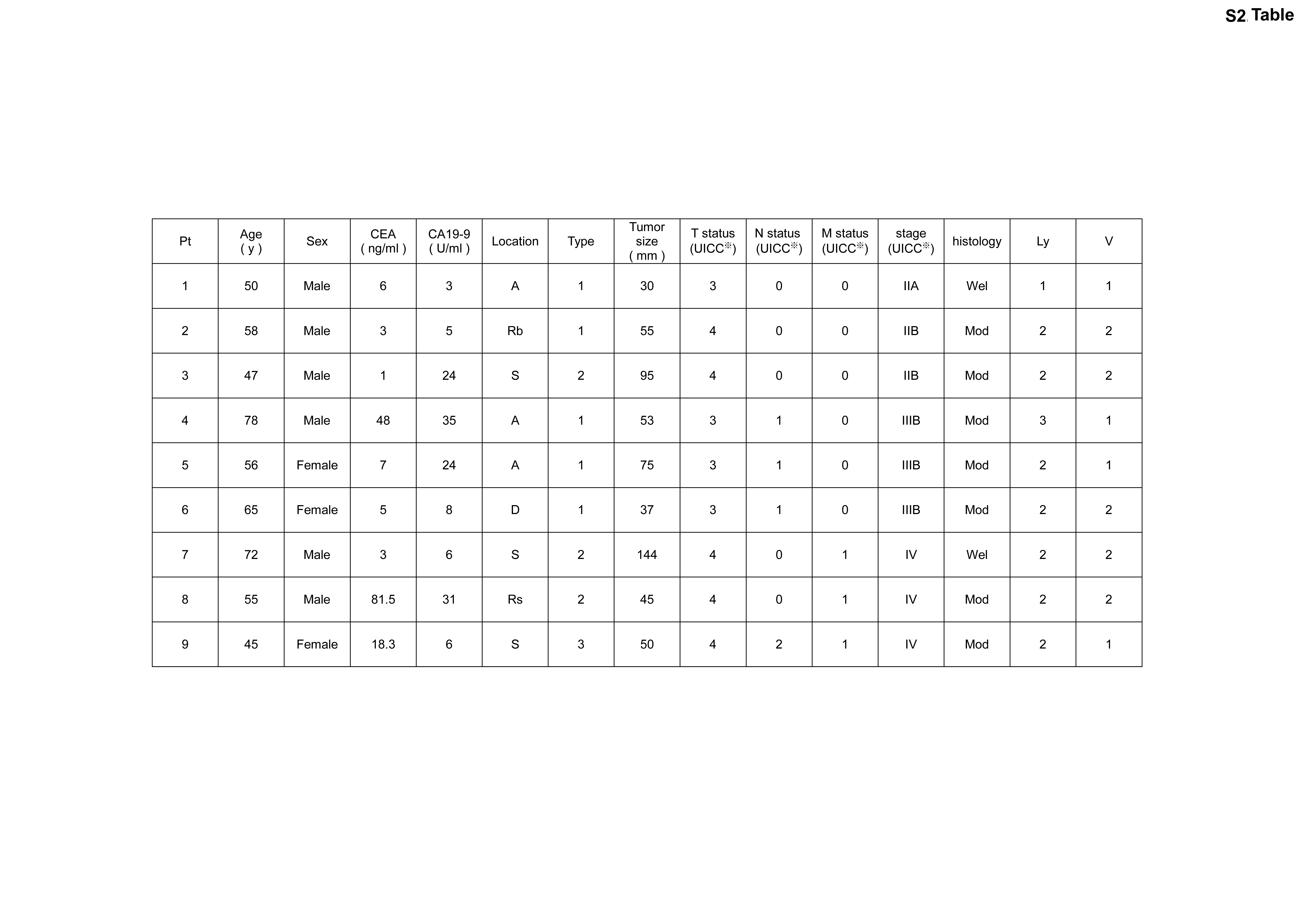

Supplement: S2 Table — Stage was according to TNM classification (UICC 7th) Abbreviations; CEA.carcinoembryonic antigen, CA19-9.carbohydrate antigen 19–9, RS. Rectosigmoid, Ra. Upper rectum, Rb.Lower rectum A. Ascending, D. Descending, S.Sigmoid, wel. well differentiated adenocarcinoma, mod. moderately differentiated adenocarcinoma. (TIFF) [file pone.0127119.s004.tiff]
